# The Green Cath Lab Challenge: Environmental Sustainability in Interventional Cardiology

**DOI:** 10.7759/cureus.103407

**Published:** 2026-02-11

**Authors:** Despoina Pappa, Maria Bourazani, Maria S Chrysi, Anna Giga, Panoraia Rammou

**Affiliations:** 1 Nursing, Henry Dunant Hospital Center, Athens, GRC; 2 Anesthesiology, Agios Savvas Hospital, Athens, GRC; 3 Surgical Nursing, Agios Savvas Hospital, Athens, GRC; 4 Nursing, 3rd Vocational High School, Athens, GRC; 5 Nursing Services and Patient Safety Consultant, Henry Dunant Hospital Center, Athens, GRC

**Keywords:** carbon footprint, cardiac catheterization laboratory, life cycle analysis, recycling, waste management

## Abstract

Healthcare systems are responsible for a significant share of global greenhouse gas emissions, and cardiac catheterization laboratories (CCLs) are among the most resource-intensive hospital units due to their reliance on single-use materials, high-energy imaging systems, and strict infection-control protocols. Understanding and mitigating their environmental impact is essential for achieving sustainable cardiovascular care. The aim of this review was to summarize and analyze available quantitative evidence on waste generation, recycling potential, and carbon emissions in interventional cardiology, and to identify practical strategies for transitioning toward a “green cath lab.” A structured literature search was conducted across PubMed, Scopus, and Web of Science for the period 2015-2025, supplemented by Google Scholar and the cardiovascular society websites. Studies were eligible if they provided quantitative data on waste output, recycling rates, or carbon footprint in CCLs. Out of 357 retrieved records, only four met all inclusion criteria. Diagnostic coronary angiography produced an average carbon footprint of 12.5 kg CO₂-equivalent per case, with single-use disposables (40%) and imaging energy (25%) as the largest contributors. Waste audits showed 1.5-3 kg of waste per procedure, 36%-40% of which was recyclable. Optimizing procedural packs, improving heating, ventilation, and air conditioning or HVAC efficiency, and structured waste segregation achieved 20%-30% reductions in waste and emissions without affecting safety. Evidence indicates that interventional cardiology generates measurable and avoidable environmental burdens. Implementing targeted, low-cost interventions can markedly reduce both waste and carbon footprint. These findings establish a quantitative foundation for sustainability frameworks, carbon auditing, and policy initiatives toward a net-zero cath lab.

## Introduction and background

Healthcare systems are responsible for an estimated 5%-10% of national greenhouse gas emissions in high-income countries, with hospitals representing a major share of that footprint [1]. Within hospitals, cardiac catheterization laboratories (CCLs) are particularly resource-intensive due to their reliance on energy-demanding imaging systems, single-use disposables, and strict infection control protocols [2,3]. These factors combine to generate large volumes of waste up to 200 kg per day in some centers and substantial electricity use from fluoroscopy and heating, ventilation, and air conditioning (HVAC) systems [4,5].

The accelerating climate crisis has positioned environmental sustainability as a central dimension of quality in modern healthcare. In cardiology, the call for "green cardiovascular care" has gained traction through major society statements and reviews emphasizing the need for carbon accounting, waste minimization, and sustainable procurement across cardiovascular services [2,3,6]. The European Society of Cardiology (ESC) and the American College of Cardiology (ACC) have both advocated integrating sustainability metrics into practice guidelines and quality programs [1,6].

Despite this increasing awareness, the environmental footprint of interventional cardiology remains poorly quantified. Early life-cycle analyses show that procedures such as diagnostic coronary angiography or percutaneous coronary intervention (PCI) carry a measurable carbon cost per case [3]. Waste audits have revealed that 30%-40% of cath lab waste could be recycled safely if appropriate segregation were implemented [7-9]. 

Heterogeneity across the included studies arises from several methodological sources, including differences in waste classification (e.g., infectious vs contaminated waste), variation in reporting denominators (per procedure, per day, or per year), and the use of different carbon conversion factors. In addition, life-cycle assessment boundaries ranged from partial (procedure-level) to broader system-based approaches, further limiting direct comparability of reported environmental impact estimates [3]. This review synthesizes the emerging evidence on the environmental impact of the cardiac cath lab, exploring the main sources of emissions and waste, summarizing practical strategies for sustainability, and discussing barriers and future opportunities in achieving a "green cath lab." By highlighting measurable interventions and identifying research gaps, this paper aims to support a structured, evidence-based transition toward environmentally responsible interventional cardiology.

## Review

Research design and data collection

This review identified and synthesized primary studies, audits, and quantitative analyses that examined waste generation, segregation, recycling, and related environmental impacts within CCLs. A structured literature search was conducted across PubMed, Scopus, and Web of Science, supplemented by Google Scholar and the official websites of major cardiovascular societies, including the ESC, the ACC, and the British Cardiovascular Intervention Society. The search covered the period from January 2015 to October 2025. Gray literature, including open-access audits and reports from the Centre for Sustainable Healthcare (UK), was also screened to ensure completeness.

Inclusion and exclusion criteria

Studies were eligible if they were peer-reviewed original research providing quantitative data on waste output, recycling potential, or carbon emissions specific to interventional cardiology practice. Exclusion criteria included review articles, editorials, opinion pieces, and studies unrelated to cardiovascular procedures. Titles and abstracts were screened independently by two reviewers, and full texts meeting inclusion criteria were analyzed for study design, setting, procedure type, waste per case (kg), recyclable fraction (%), and estimated carbon footprint (kg CO₂-equivalent per procedure).

**Figure 1:**
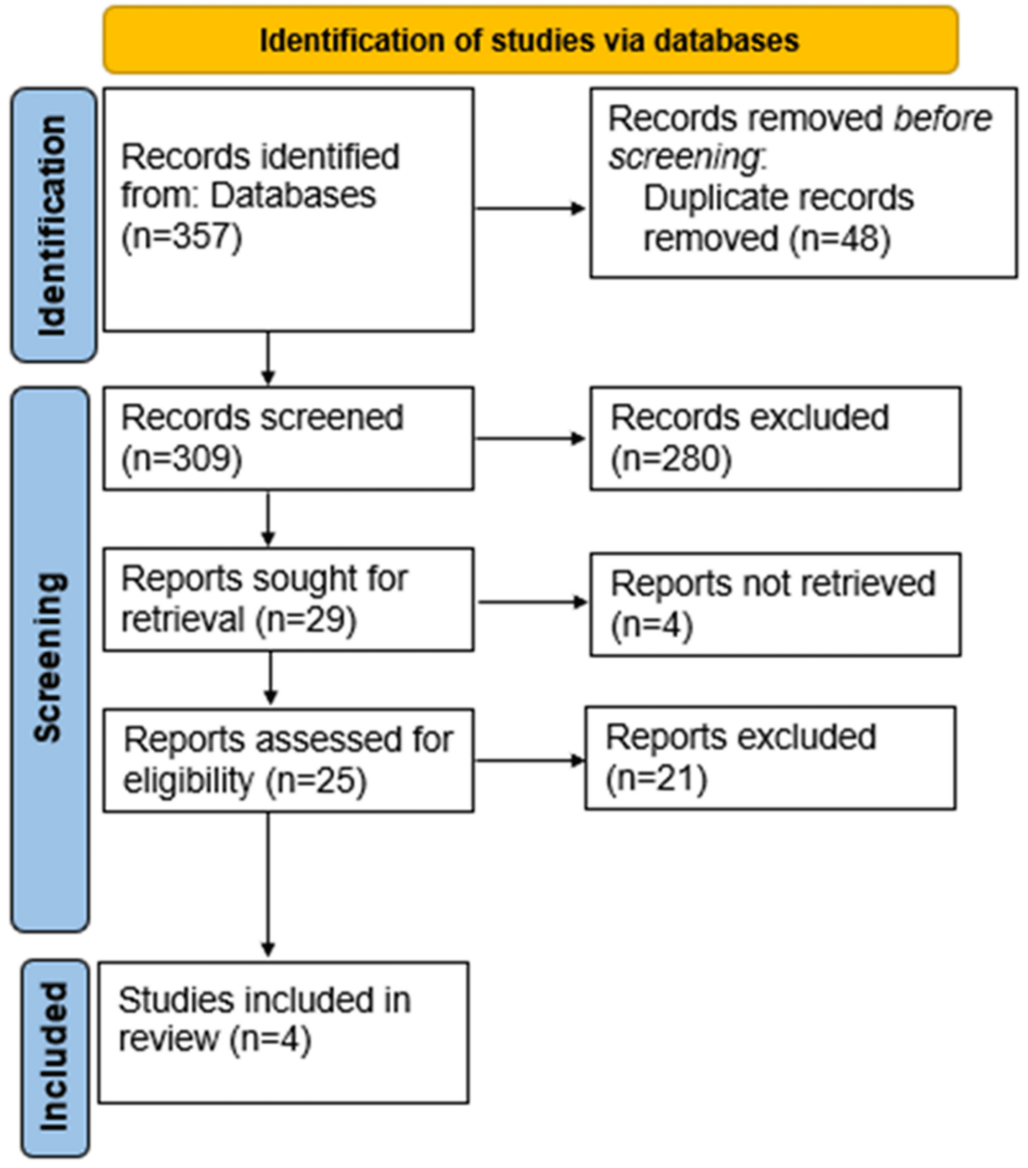
PRISMA flow diagram PRISMA, Preferred Reporting Items for Systematic reviews and Meta-Analyses.

Out of an initial pool of 357 records, only four studies ultimately met all inclusion criteria and provided complete, procedure-specific quantitative data: the sustainable practices of Chen et al., in 2025 [10], the life-cycle analysis (LCA) by Leiszt et al., in 2025 [11], the observational audit by Doshi et al., in 2023 [6], and the multicenter audit by Amin et al., in 2024 [5] (Figure 1). These four studies formed the empirical foundation of the results section, while additional descriptive sources were used for contextual discussion.

Due to heterogeneity in study designs, metrics, and reporting methods, no statistical meta-analysis was performed. Instead, data were synthesized narratively to highlight shared trends, quantitative ranges, and actionable opportunities for sustainability in interventional cardiology. The present review did not undertake a formal assessment of selection or measurement bias, nor a standardized evaluation of system boundary assumptions used in life-cycle assessments. As a result, some variability in reported environmental impact estimates across studies should be interpreted with appropriate caution.

Results

The literature research identified a limited but growing body of quantitative evidence on waste generation and environmental impact in CCLs, with four primary studies providing detailed empirical data (Table 1). Chen’s, Leiszt’s, Doshi’s, and Amin’s studies were chosen among 357 records. Collectively, these investigations demonstrate that interventional cardiology procedures generate a measurable and preventable environmental footprint primarily driven by single-use consumables, energy-intensive imaging systems, and suboptimal waste segregation practices.

**Table 1:** Summary of primary studies included in the review HVAC, heating, ventilation, and air conditioning; USA, United States of America.

Author (year)	Country/setting	Study design	Procedures or sample	Main findings	Key conclusions
Chen et al., 2025 [10]	Taipei, Taiwan	One-year interventional approach	Waste output measurement at four time points	Infectious waste: 2.38 → 1.54 kg/procedure (−35.4%). General waste: 0.83 → 0.61 (−26.4%). Recyclable paper: +55.5%. Annual CO₂-eq reduction: 1,084 kg	The year-long intervention complements the one-week and single-procedure observations of the previous three studies, demonstrating sustained, system-level waste reductions.
Leiszt et al., 2025 [11]	Italy (University Hospital of Catania)	Life-cycle analysis of diagnostic coronary angiography	Single diagnostic coronary angiography	Mean carbon footprint: 12.5 kg CO₂-eq per case. Major contributors: disposables 40.6%, imaging 25%, and HVAC 20%. Optimization (energy + procedural packs) reduced footprint to 9.8 kg CO₂-eq (−22%).	Diagnostic coronary angiography has a quantifiable CO₂ footprint; small workflow and energy efficiency interventions can yield meaningful emission reductions.
Doshi et al., 2023 [6]	USA (tertiary cardiac catheterization lab)	Observational waste audit (five days, 70 cases)	Diagnostic angiography and primary coronary intervention (PCI)	Average recyclable waste: 0.72 kg per diagnostic case, 1.41 kg per PCI. 80% of recyclables were plastic/paper. Structured segregation could divert ≈0.7 tons/1,000 procedures from landfill and cut costs by 20%-25%.	Cath lab waste is largely non-infectious and recyclable; segregation and recycling programs can significantly reduce environmental and financial burden.
Amin et al., 2024 [5]	Bahrain and Switzerland (multicenter)	Retrospective audit of recyclable vs contaminated waste	Annual output from invasive cardiac procedures	11,000 kg of recyclable and 30,000 kg of contaminated waste/year. ≈2.3 kg waste per procedure; 36%–40% recyclable. Global extrapolation: >150,000 tons of potentially recyclable waste annually	A large portion of cath lab waste can be safely recycled; structured waste management could halve incinerated material globally.

In the first comprehensive LCA, Leiszt et al. quantified the greenhouse gas emissions associated with a standard diagnostic coronary angiography, reporting an average carbon footprint of 12.5 kg CO₂-equivalent per case [11]. Approximately 40% of emissions stemmed from disposable materials, 25% from imaging system electricity consumption, 20% from HVAC and lighting, and 9% from waste transport and treatment. Implementing optimized procedural packs and improved HVAC cycling reduced emissions by roughly 22% (down to ~9.8 kg CO₂-equivalent) [11].

The study by Chen et al., in 2025, evaluated the impact of a comprehensive, year-long sustainability intervention implemented in a high-volume CCL performing 2,807 procedures annually [10]. The intervention combined staff education, the establishment of a dedicated “green team,” continuous waste monitoring, improved segregation between infectious and general waste, and expansion of recycling programs. The results demonstrated substantial and sustained reductions in both waste generation and carbon emissions. Infectious waste decreased from 2.38 kg to 1.54 kg per procedure, representing a 35.4% reduction, while general waste fell from 0.83 kg to 0.61 kg per procedure (26.4% reduction) [10].

Recycling performance improved notably: paper recycling increased by 55.5%, and plastic recycling by 16.2%, indicating a significant shift toward more sustainable waste management. When extrapolated annually, these improvements produced a total reduction of 1,084.2 kg CO₂-equivalent, confirming meaningful environmental benefit. Overall, the study shows that a structured, multicomponent intervention - requiring minimal financial investment - can achieve long-term, system-level improvements in waste reduction, recycling efficiency, and carbon footprint in a busy catheterization laboratory, without compromising workflow, procedural efficiency, or patient safety [10].

In parallel, Doshi et al. conducted a five-day observational audit in a US tertiary cath lab involving seventy procedures, quantifying an average of 0.72 kg of recyclable material per diagnostic case and 1.41 kg per PCI, with total recyclable waste reaching 52.6 kg during the sampling period: plastics and paper packaging accounted for more than 80% of the recyclable mass. Extrapolation suggested that structured segregation could divert ≈0.7 tons of landfill waste per 1,000 procedures and decrease disposal costs by up to 25% [6].

Similarly, Amin et al. performed a multicenter audit across invasive cardiac units in Bahrain and Switzerland, documenting an annual output of ~11,000 kg recyclable waste and ~30,000 kg contaminated waste, corresponding to roughly 2.3 kg total waste per procedure, with 36%-40% safely recyclable [5]. The authors estimated that if such proportions were replicated globally, invasive cardiology could generate over 150,000 tons of potentially recyclable material each year. When these findings are viewed collectively, they reveal that CCLs typically produce between 1.5 and 3 kg of waste per procedure, of which about 40% could be diverted from incineration or landfill, while diagnostic angiography alone emits around 10-13 kg CO₂-equivalent, and percutaneous interventions roughly double that figure.

The convergence of data from different continents underscores a common pattern: disposable-dominated workflows and lack of formalized recycling programs represent the main environmental burdens. Conversely, all studies highlight that relatively simple interventions - such as optimizing procedure packs, improving waste segregation, and rationalizing energy use - can collectively achieve 20%-30% reductions in both waste and emissions without compromising patient safety or procedural efficiency. These results underscore the feasibility and clinical compatibility of “green cath lab” strategies and establish a quantitative foundation for future multicenter benchmarking and carbon-reduction initiatives in interventional cardiology.

Discussion

The emerging body of quantitative evidence demonstrates that the CCL is a significant contributor to the environmental footprint of modern cardiovascular medicine. The four original studies forming the empirical core of this review - Amin’s, Doshi’s, the year-long quality-improvement initiative by Chen et al., and Leiszt’s - provide converging data that interventional cardiology generates measurable greenhouse gas emissions and large volumes of potentially recyclable waste. Their findings, supported by broader conceptual analyses of cardiovascular sustainability, point to a clear conclusion: the transition toward a “green cath lab” is both feasible and urgently needed [5,6,10,11].

Leiszt et al. performed the first comprehensive LCA of a diagnostic coronary angiography. The study quantified a mean carbon footprint of 12.5 kg CO₂-equivalent per case, distributed among single-use consumables (40.6%), energy consumption for imaging and HVAC systems (45%), and waste transport/treatment (≈9%). By implementing optimized procedural packs and improved energy management, the total footprint fell to ≈9.8 kg CO₂-equivalent, demonstrating that even small workflow changes can yield >20% emission reduction [11]. This work provides a quantitative baseline for carbon accounting in CCLs.

Complementing this, Doshi et al. conducted a five-day observational audit of 70 procedures in a US tertiary CCL. They measured 0.72 kg of recyclable waste per diagnostic case and 1.41 kg per PCI, identifying plastics and paper as the dominant components (>80%). The authors estimated that structured waste segregation could divert ≈0.7 tons of landfill waste per 1,000 procedures and lower disposal costs by 20%-25% [6]. Their work highlights the tangible economic co-benefits of sustainability initiatives.

The year-long quality-improvement study by Chen et al. adds an important longitudinal and implementation science dimension to this evidence base [10]. In a high-volume CCL performing 2,807 procedures annually, stepwise interventions - centered on staff education, routine tracking of waste metrics, improved waste segregation, the creation of a sustainability team, and regular feedback - produced sustained reductions of 35.4% in infectious waste and 26.4% in general waste, alongside a 55.5% increase in recyclable paper and a 16.2% increase in overall recyclables. The estimated annual reduction in carbon emissions was 1,084.2 kg CO₂-equivalent, with the largest contribution arising from decreases in incinerated infectious waste. Importantly, these improvements were achieved without altering clinical protocols or compromising patient safety, and were maintained over one year, illustrating that sustainability initiatives can be embedded into routine operations rather than remaining short-lived “pilot” projects [10]. This study therefore provides a practical framework - spanning pre-operative, intra-operative, and post-operative phases that other CCLs can adapt to their local context. Viewed in a broader context, these findings align with system-level analyses of cardiovascular care and healthcare sustainability. Barratt et al. showed that cardiovascular services are among the more emission-intensive domains of hospital activity while also highlighting substantial methodological heterogeneity and a lack of standardized metrics across existing environmental assessments [12].

The multicenter audit by Amin et al. broadened these observations to two international centers, reporting an annual production of ~11,000 kg recyclable and 30,000 kg contaminated waste, equivalent to 2.3 kg per invasive procedure with 36%-40% recyclable potential. Extrapolated globally, this corresponds to more than 150,000 tons of recyclable material per year from invasive cardiology alone. Collectively, these audits reveal that the majority of procedural waste is non-infectious and could be safely diverted from incineration if appropriate segregation systems were established [5].

The broader interpretative context provided by Rajagopalan and Alasnag studies places these findings within a systems-level framework [1,2]. Rajagopalan et al. emphasize that cardiovascular care contributes disproportionately to hospital emissions due to the energy-intensive nature of imaging and the dominance of disposable technology [1]. Alasnag et al. outlined a model of cath lab sustainability that integrates waste management, energy efficiency, supply chain accountability, and staff engagement, principles directly reflected in the quantitative results of the three primary studies. Their work underlines that sustainability is not peripheral to quality but an intrinsic component of patient-centered, cost-effective care [2].

Al-Kindi et al. further argue for institutional and policy-level transformation. They propose embedding environmental metrics - such as waste output, CO₂ intensity per procedure, and energy use - into accreditation frameworks and procurement processes [13]. Linking these metrics to reimbursement or quality incentives could accelerate behavioral change at scale. The authors also advocate collaboration between manufacturers and clinicians to redesign device packaging and enable circular supply chains, thereby reducing upstream emissions that lie outside the direct control of clinical teams [13].

Despite the encouraging progress, the evidence base remains limited. All four primary studies are single-center or short-term audits, and heterogeneity in methodology - different denominators, system boundaries, and conversion factors - limits cross-study comparability. There is also a lack of standardized carbon accounting tools tailored for interventional cardiology, as well as regulatory uncertainty regarding the safe re-use of devices. Future research should focus on multicenter LCAs with harmonized methods and inclusion of economic analyses quantifying cost savings alongside emission reduction. Within the cath lab itself, narrative and consensus pieces provide concrete strategies that resonate with the empirical results of the present review. Szirt et al. described the “5R” framework - reduce, reuse, recycle, rethink, and research - and illustrated how lean procedural packs, selective opening of devices, and greater reliance on non-invasive testing where appropriate can decrease both material consumption and waste generation [14]. A recent scoping review of sustainability and waste reduction strategies in CCLs suggests that most published interventions are not only feasible, inexpensive, and compatible with existing clinical workflows but that outcome reporting is inconsistent and often limited to short-term case series [15].

Beyond interventional cardiology, life-cycle assessments of coronary artery bypass grafting and other invasive procedures in surgery and interventional radiology confirm that resource-intensive procedural pathways carry substantial environmental burdens, driven by single-use consumables, anesthetic gases, and perioperative energy use [7,16].

Methodological work on healthcare LCA and open databases such as HealthcareLCA provide standardized approaches and reference values that can be adapted to CCL settings, enabling more robust comparisons across institutions and over time. However, current CCL studies rarely capture upstream emissions from device manufacturing and logistics, or downstream impacts such as patient travel and follow-up imaging, implying that the true carbon footprint of interventional cardiology is likely underestimated [1].

A further methodological limitation of the present review is the absence of a formal or semi-formal assessment of selection bias, measurement bias, and system boundary assumptions within the included studies. In particular, substantial heterogeneity was observed in life-cycle assessment boundaries, data sources, and emission-conversion factors, which may lead to under- or overestimation of reported carbon footprints and limit direct comparability across studies. These factors should be considered when interpreting the findings and highlight the need for future research adopting standardized bias assessment tools and harmonized LCA frameworks tailored to interventional cardiology.

Nevertheless, the converging data show that meaningful improvement is feasible now. Integrating staff education, structured waste segregation, and optimized energy use could reduce emissions and waste by 20%-30% without compromising safety or efficiency. The evidence also highlights that environmental stewardship can align with financial sustainability, reducing waste processing costs and material procurement expenses. In line with Rajagopalan and Al-Kindi studies, these findings affirm that sustainability must become an integral element of cardiovascular care quality standards [1,13]. The “green cath lab” is therefore not a distant aspiration but an achievable, evidence-based evolution of contemporary interventional cardiology.

## Conclusions

Current evidence shows that practical, low-cost interventions can significantly reduce waste and carbon emissions in the CCL without compromising clinical care. However, despite the promising results from existing studies, the field remains limited by the small number of robust primary investigations. To advance sustainable practice, more well-designed, multicenter primary studies are needed to quantify environmental impact across procedures and to evaluate long-term, scalable interventions. Strengthening this evidence base will be essential for integrating environmental sustainability into routine cardiovascular care.
